# Health literacy among self‐help leprosy group members reduces stereotype endorsement and stigma‐related harm in rural Nepal

**DOI:** 10.1111/hsc.13771

**Published:** 2022-02-27

**Authors:** Orla T. Muldoon, Sarah Jay, Aisling T. O'Donnell, Michael Winterburn, Andrew B. Moynihan, Brenda H. O'Connell, Ramesh Choudhary, Kiran Jha, Arbind K. Sah

**Affiliations:** ^1^ 8808 Centre for Social Issues Research University of Limerick Limerick Ireland; ^2^ Nepal Leprosy Trust Lalgadh Nepal; ^3^ 8810 Computing and Informatics Research Group Limerick Institute of Technology Limerick Ireland; ^4^ 8798 Department of Psychology Maynooth University Maynooth Ireland

**Keywords:** leprosy, social cure, stereotype endorsement, stigma

## Abstract

There is increasing appreciation that group memberships can have both beneficial and damaging impacts on health. In collaboration with Nepal Leprosy Trust (NLT), this longitudinal study explores a group‐based approach to stigma reduction among people affected by leprosy in rural Nepal (*N* = 71)—a hard to reach and underrepresented non‐WEIRD population. Informed by the ‘social cure’ literature, and the progressive model of self‐stigma, we use a longitudinal design. We found that a sense of belonging to a self‐help group can facilitate education in terms of health literacy, and over time these two factors also have impacts on participants stigma. Specifically, self‐help group belonging predicted improvements in health literacy, leading to reduced endorsement of negative stereotypes and thus less stigma‐related harm among people affected by leprosy. The study offers promising evidence that group‐based interventions, which support health education, can reduce the harmful impact of stigma in very challenging contexts.

**What is known about this topic?**

- Leprosy remains a highly stigmatised condition.
- Stigma can interfere with diagnosis and treatment of health conditions such as leprosy.
- Group memberships can benefit a health in a phenomena labelled ‘the social cure’.

**What this paper adds?**

- A sense of belonging to a leprosy self‐help group enhances health literacy which led to reduced self‐stigma amongst self‐help group members.
- Self‐help group membership is a social cure for the stigma‐related harm amongst those affected by leprosy.
- Support for the ‘social cure’ model and, and the progressive model of self‐stigma in a non‐WEIRD (Westernised, Educated, Industrialised, Rich Developed) context.

## 1 INTRODUCTION

In this paper, we present a longitudinal community study of those affected by a disease that has been stigmatised since biblical times, so much so that leprosy has become synonymous with stigma. Our study, with rural village dwellers in Nepal, is informed by two models for understanding and addressing stigma‐related harm in community settings. First, informed by the social cure approach (Jetten et al., 2012), we examine whether a sense of belonging to a self‐help group, ostensibly teaching disease management and general health literacy, can counteract the marginalisation and exclusion felt by those affected by leprosy. Second, informed by the progressive model of self‐stigma (Corrigan et al., 2011), we examine the potential for any improvements in health literacy to reduce stigma‐related harm. In this way, we hope to test two models for tackling stigma in a nonWEIRD (Westernised Educated Industrialised Rich Developed) community setting.

Research in the tradition of the social cure points to the value of connections and sense of group belonging to health promotion (Wakefield et al., 2013). The social cure model suggests that group memberships are associated with positive outcomes in a range of health contexts, including stigmatising ones, for example in determining healthy ageing (Haslam et al., 2014), recovery from addiction (Best et al., 2016) and adjustment to acquired brain injury (Muldoon et al., 2019; Walsh et al., 2015). A sense of belonging to a group (Greenaway et al., 2016) has been presented as the important ingredient driving these social cure effects (Cruwys et al., 2014). A primary aim of this research therefore is to examine the potential for a self‐care group to counteract some of the exclusion felt by those affected by leprosy by offering an improved sense of belonging.

Despite evidence in support of the social cure, there are occasions where group memberships, most notably stigmatised group memberships, can damage health. Corrigan et al. (2011) propose a progressive model of self‐stigma, whereby those affected by a stigmatised condition are aware of the stereotype relating to their group, may then also agree with it, apply it to themselves, and as a result can suffer harm. The degree to which people who are stigmatised apply negative stereotypes to themselves has also been termed stereotype endorsement (Ritsher et al., 2003), and thus forms one aspect of harmful internalised stigma (Corrigan & Watson, 2002; Corrigan et al., 2006). In this study, then, we consider whether self‐help groups can ameliorate some of the negative internalisation of leprosy stigma by altering these two stigma processes—namely, stereotype endorsement and stigma‐related harm.

In Corrigan et al. (2011) progressive model of self‐stigma, this *application* or stereotype endorsement stage is the most proximal aspect of self‐stigma to harm. Indeed, Corrigan and colleagues note that each stage of this model is most affected by the one immediately preceding it, so it is perhaps unsurprising that application is the most psychologically harmful stage (Corrigan et al., 2006, 2011; Ritsher & Phelan, 2004; Weiss, 2008). If stereotype endorsement can be reduced through the skills and knowledge gained from a self‐help group intervention, then theory and prior evidence would suggest that the harm associated with self‐stigma can be reduced as well. In the present study, then, we hypothesise that when stereotype endorsement can be reduced, the harm associated with self‐stigma can also be reduced.

It is notable that these aspects of leprosy stigma are the same as those applied to people living with HIV/AIDS (Audet et al., 2013). Yet, despite this detailed typology that is supported by broader stigma literature, most literature on leprosy stigma does not attend to the perceptions, fears, and internalised views of the person with leprosy, focusing instead on participation in the community (Van Brakel, 2003). An exception is Dadun et al. (2017), who orient to several aspects of leprosy stigma including internalised or self‐stigma. We argue this is a vitally important development, as the perspective of the person who is affected by leprosy is not yet fully understood (Adhikari et al., 2014). Building on this, there is the potential to develop theoretically grounded research into self‐stigma associated with leprosy, and the extent to which this may be reduced by interventions such as self‐help groups.

The negative stereotypes linked to leprosy stem from social segregation, low Caste position and the associated extreme poverty and social disadvantage of those affected (Van Brakel & Miranda Galarza, 2013). They are also linked to poor health literacy and education that pervade in these vulnerable groups. For many, leprosy is still not seen as a communicable bacterial disease, rather than a punishment for past sins (Cross et al., 2011). Group‐based interventions can be used to explicitly target leprosy‐specific and general health literacy and at the same time facilitate social connections that are likely to be particularly important for tackling stigma and the endorsement of negative self‐stereotypes.

### 1.1 The present study

This paper reports on a study of people affected by leprosy in rural Nepal, who on being diagnosed were offered group‐based community support to improve their leprosy self‐care and general health literacy. To minimise attrition in our studies in these hard to reach rural populations, two surveys were conducted 6 months apart in May/June and November/December 2017. Drawing on the theoretical evidence reviewed, it was hypothesised that participants in the self‐help groups would show an increase in their sense of belonging to the group (H_1_) as the aim of the self‐help group was improved health literacy (H_2_). We also anticipated reductions in stereotype endorsement (H_3_) and stigma‐related harm (H_4_) across time. Secondly, as increases in belonging motivates health and social engagement (Haslam et al., 2014; Kitchen et al., 2012), belonging was hypothesised to predict improvements in health literacy across time (H_5_). Next, in line with the progressive model of self‐stigma (Corrigan et al., 2011), improved health literacy was hypothesised to reduce stereotype endorsement and stigma‐related harm from Time 1 to Time 2 (H_6_). Integrating the social cure and progressive model of self‐stigma, we then tested whether change in sense of belonging and health literacy across time predicted stereotype endorsement and stigma‐related self‐harm at Time 2 (H_7_).

## 2 METHOD

### 2.1 The community context

Participants were members of 10 self‐help groups who individually consented to participate in the research. This included 7% of all groups being facilitated at that time by the Nepal Leprosy Trust (NLT) in ten villages in four districts of Southern Nepal: Dhanusha, Mahottari, Sarlahi and Sindhuli at that time. Villages were selected to represent a range of cultural and caste groupings and to include those that had new and established links with NLT. The population served by groups operated from NLT’s Lalgadh Leprosy Hospital and Services Centre (LLHSC). It is located in Province 2 of Nepal and in a geographical region known as the Terai, which ranks lowest in Nepal's National Human Development Indicators. It scores more poorly than other regions of Nepal on indicators such as employment, life expectancy, literacy, maternal mortality (Nepal Human Development Report, 2020). The ‘Nepali’ identity is traditionally considered a culture of hill‐dwelling high Chhetri‐Brahmin castes (Acharya & Muldoon, 2017). Most residents of the Terai, where Leprosy remains a pressing health concern, do not understand and speak the Nepali language affecting access to education and resources within the state. Residents of the region have also been denied citizenship rights.

This region has some of highest rates of leprosy prevalence and disability in South Asia, at approximately 2.76 per 10,000 population in 2019 compared with 2015 global prevalence of 0.18 per 10,000 population. The elimination and treatment of leprosy has always been a priority of the Government of Nepal. NLT and LLHSC support the National Leprosy Control Programme (NLCP) by strengthening the leprosy referral and specialised hospital services. Although Nepal had achieved leprosy elimination status, the challenges of sustaining this 7–8 achievement remain. The latest WHO statistics (2019) shows Nepal is again above the 1/10,000 prevalence, and elimination has failed. Barriers that impede the adherence and treatment include stigma, health literacy, poverty, long distances and costs to access services, and reliance on local traditional healers.

### 2.2 Measures

Participants were asked to respond to all measures on a five‐point scale. To aid comprehension, a picture was provided at the beginning of the questionnaire. The picture format developed for use with populations with limited literacy (Tewari et al., 2012) and previously used in Nepal (Muldoon et al., 2017) showed five glasses with increasing water volume, representing empty (0 = *Not at all*) to full (4 = *Extremely/always*).

#### 2.2.1 Stereotype endorsement

Stereotype endorsement was measured with three items of Ritsher et al. (2003) scale. These three items are the stereotype endorsement subscale of a multidimensional measure, which has five subscales. Examples of items include ‘Most of the times, I need someone to make my decisions because I have leprosy’ and ‘I cannot contribute anything to the society because I have leprosy’. Mean scores across the three items created a total score with higher scores indicating higher levels of stereotype endorsement. The scale reliability was adequate: alpha coefficient Time 1 α = 0.60 and Time 2 α = 0.76.

#### 2.2.2 Stigma‐related harm

Stigma‐related harm was measured with four items from Dadun et al.’s (2017) Stigma Assessment Reduction Impact (SARI) scale, a cross culturally validated instrument to measure leprosy‐related stigma. All four items were drawn from the internalised stigma subscale. Sample items include ‘Do you blame yourself for being affected by leprosy?’ and ‘Have you regretted telling someone that you have leprosy?’ Mean scores across the four items created a total score with higher scores indicating higher levels of stigma‐related harm. The scale reliability was good: alpha coefficient Time 1 α = 0.70 and Time 2 α = 0.76.

#### 2.2.3 Self‐help group belonging

Self‐help group belonging was measured with three items adapted from Leach et al. (2008) measure of in‐group identification. This is a self‐report subjective measure of people's experience of in‐group belonging. A sample item includes ‘I feel that I am similar to other members of the self‐help group’. Mean scores across the three items created a total score, with higher scores indicating higher levels of self‐help group belonging. The scale reliability was good; at Time 1 α = 0.83 and at Time 2 α = 0.76.

#### 2.2.4 Health literacy

Health literacy was measured with an adapted version of Lee et al. (2010) Short Assessment of Health Literacy. The test included nine word association items. Participants read nine health terms and are asked to associate each term to another word, to demonstrate comprehension. So for example, respondents would be awarded a score of 1 if they correctly associated infection with fever or kidney with urine. A score of 0 was awarded for incorrect or do not know response. The scale reliability was adequate: Cronbach's alpha at Time 1 α = 0.75 and Time 2 α = 0.71.

### 2.3 Procedure

The survey was compiled in English and discussions about cultural appropriateness of the items within the team followed. One native Nepali speaker first translated the survey from English to Nepali, and the second back translated it from Nepali to English. The English version was checked for accuracy, and where necessary, adjustments were made to the Nepali version.

On the day prior to commencement of Time 1 data collection, a 1‐day training workshop was delivered in Nepal, in English and Nepali, by the first, second and fourth authors to 12 community team workers at Lalgadh hospital, who then acted as research assistants. This opportunity was used to increase the research capital within the community health care team in Lalgadh. Conscious of power differentials, the value of both hypothesised and null findings to research processes were reviewed extensively to minimise potential biases. A refresher session was held at Time 2 and data collection occurred in rural villages across the following 2 weeks.

Data collection was divided between two teams, with one team working in one or two villages, time permitting, on a given day. Research assistants explained the study and invited self‐help group members to participate. Once the study was explained and consent obtained, the team member and participant walked to a quiet place away from others to complete the survey. As the majority of participants were not literate, surveys were read aloud to participants, with female participants being matched with a female research assistant where at all possible. Although the surveys were printed in Nepali, the community team had to verbally translate them into either Maithili or Tharu, which are local languages, and in some instances Hindi. The surveys took on average 30 min to complete.

### 2.4 Ethical issues

All participants were asked for full consent to participate in the study which had full prior ethical approval from the University ethics committee. Where participants were illiterate and consent was indicted using a thumb print. There are many ethical issues with this study. The power imbalance between participants, community team workers and white English‐speaking researchers in this instance is compounded by the fact that self‐help group members are some of the poorest and most marginalised members of Nepali society, who are in receipt of health and welfare services from the community teams. Considerable time was devoted during the training workshop to talk about this power imbalance and potential for demand characteristics. Five of the authors were both new and external to NLT, which we hope facilitated impartiality. Four of the authors, who were actively involved in the management of data collection, had long standing links with NLT. The team viewed the project as an important opportunity to increase research capital within the organisation. The potential for those assisting with data collection to inadvertently influence findings and the need to guard against these biases was emphasised in training before data collection as was the value of both null effects to evidence base, particularly where resources are limited.

The emphasis during training of researchers who went into the field, community teams understood the importance of voluntary participation and the possibility of social desirability and experimenter effects in participant responses. Low literacy levels meant participants had less privacy and less control over their responses than if they were able to read the items themselves. On approaching participants to invite their contribution, they were assured that partaking was voluntary, and that they could withdraw at any time. Participants were also informed clearly and unequivocally that the services they receive from the hospital and community team did not depend on their participation in the study. Once this was understood, participants signed the consent form or used an ink pad to provide a thumb print to demonstrate their individual consent to participate. Interest in the study, as it was an unusual occurrence, was very high, as was consent to participate. However, participants were advised to answer only those questions that they were willing to answer and so there are some missing data points where participants chose not to answer (See also Jay et al., 2021 for discussion of potential empowerment from study participation).

### 2.5 Approach to analysis

IBM SPSS Statistics 23 was used to conduct analysis, with α = 0.05 as criterion for significance. Due to our limited sample size, we began by conducting a sensitivity analysis using G*Power. This analysis showed that based on our sample size, an alpha value of 0.05 and 80% power had capacity to detect medium effects (*f* = 0.085). To test hypothesis 1–4, repeated measures ANOVAs were conducted to examine change in self‐help group belonging, health literacy, stereotype endorsement and stigma‐related harm, across time. Subsequently, the relationships between these variables, at and across Time 1 and Time 2, were examined using simple bivariate correlations. Additional ANOVAs and partial correlations were conducted to ensure the pattern of relationships held across gender and ethnic groups. The PROCESS macro for SPSS was used to test hypothesis 5–7 using serial mediation. Each participant's score on self‐help group belonging at T1 and health literacy at T1 were subtracted from their respective T2 scores. The serial mediation model then used these scores to test whether change in self‐help group belonging predicted reduced stigma‐related harm at T2, controlling for stigma‐related harm at T1. We tested if changed health literacy and T2 stereotype endorsement acted as mediators of the link between belonging and harm. Results are reported for bootstrap significance tests using a bias‐corrected and accelerated (BCa) 95% confidence interval (CI) and employ a resample procedure of 5000 bootstrap samples.

## 3 RESULTS

### 3.1 Participants

The participants in these studies were people affected by leprosy encouraged and supported by the Lalgadh hospital community team to establish self‐help groups in which participants learn about and support each other in practices aimed at promoting self‐care to reduce skin ulceration, non‐formal education to promote improved inclusion and health literacy. Our participants represent some of the very poorest people living in rural Nepal. Participants had limited primary education or no education at all. Only 7% of our sample was literate (see Table 2) Family incomes were reported to be approximately 112,030.86 Nepali rupees or €781 or $945 per annum placing our sample in the poorest quintile in Nepal (Central Bureau of Statistics, 2011). The majority (*n* = 63 of *N* = 71) of our participants were Madhesi and Dalit. Madheshi communities have historically been discriminated against as second‐class citizens within Nepal. Dalit, the term used to refer to the formerly ‘untouchable castes’, is a religiously, culturally, economically and socially marginalised group.

The study reports longitudinal data collected at two time points 6 months apart. Of the 98 people who participated at Time 1, there were 71 remaining at Time 2, with a 27.6% drop out rate. To test for bias in the remaining sample, independent samples *t*‐tests were conducted (see Table 1). There were no significant differences at Time 1 between those who remained in the study and those who dropped out among our key variables. The final sample of 71 participants is described in Table 2, including ethnic/caste group information.

**TABLE 1:** Independent samples *t*‐tests showing no differences between those who remained and those who dropped out of the study on key variables

	Remained T1 Mean (*SD*)	Dropped out T1 Mean (*SD*)	*t*	*df*	*p*
Stereotype endorsement	1.53 (1.11)	1.48 (0.83)	0.23	96	0.82
Stigma‐related harm	1.23 (0.96)	1.51 (1.0)	1.29	96	0.20
Health literacy	5.82 (1.9)	5.44 (1.8)	0.88	92	0.38
Self‐help group belonging	3.84 (0.30)	3.75 (0.38)	1.10	96	0.30

**TABLE 2:** Participant information

Variable	Participants		
	*N*	%	Mean (*SD*)
Age			55.40 (16.03)
Gender			
Male	44	63	
Female	25	36	
Did not disclose	2	1	
Marital status			
Married	63	89	
Single	8	11	
Leprosy status			
Currently affected	10	15%	
Affected in past	61	85%	
Family member affected by leprosy			
Yes	14	20	
No	57	80	
Disability due to leprosy			
Visible	28	40	
Non‐visible	23	32	
None	20	28	
Education			
None	36	50	
Primary school only	19	27	
Literate	5	7	
Did not declare	11	16	
Family income			112,030.86 Nepali rupees (118,860.48) (Equivalent to €877.00)
Ethnicity			
Khas Dalit	3	4.23	
Madheshi Dalit	32	45.07	
Janajati	3	4.23	
Madheshi high caste	2	2.81	
Madheshi other	26	36.62	
Khas other	4	5.63	
Did not specify	1	1.41	

### 3.2 Changes in measures across time

All measures were taken at two time points, approximately 6 months apart, during which time our leprosy affected participants had attended weekly self‐help group meetings facilitated by the community health worker affiliated to the Nepal Leprosy Trust.

In support of hypotheses 1–4, repeated measures ANOVA indicated that scores on the health literacy measure increased significantly between T1 (*M* = 5.91) and T2 (*M* = 6.55). There were also significant differences in both stereotype endorsement between T1 (*M* = 1.53) and T2 (*M* = 0.69) and stigma‐related harm at T1 (*M* = 1.21) and T2 (*M* = 0.58). Self‐help group belonging scores were at the upper limits of the response scale T1 (*M* = 3.84) and T2 (*M* = 3.82), and did not change significantly. Please see Table 3 for all ANOVA results.

**TABLE 3:** Repeated measures ANOVA results showing changes in key variables from time 1 to time 2

	*F* (*df*)	*p*	η_p_^2^
Self‐help group belonging	0.21 (1, 70)	0.65	0.003
Health literacy	5.18 (1, 66)	0.026	0.07
Stereotype endorsement	28.50 (1, 69)	<0.001	0.29
Stigma‐related harm	23.34 (1, 69)	<0.001	0.25

Note

Differences in degrees of freedom are caused by missing values on certain variables.

### 3.3 Relationships across time

There were significant relationships between age and income and several of our measures. Those with lower incomes reported greater feelings of belonging to their self‐help group at T1 (*r* = −0.33, *p* = 0.01) and T2 *(r* = −0.31, *p* = 0.01) and lower T2 health literacy (*r* = −0.33, *p* = 0.01). Older people reported more T1 stigma‐related harm (*r* = 0.22, *p* = 0.02), and lower T1 health literacy *(r* = −0.25, *p* = 0.01) and lower T2 stereotype endorsement (*r* = 0.44, *p* = 0.01).

Table 4 presents the cross‐sectional relationships between the study variables measured at and across Time 1 and Time 2. As expected, stereotype endorsement and stigma‐related harm were positively related at T1 and at T2. Higher levels of each of these were also linked to reports of lower T1 health literacy and increased T2 self‐help group belonging. Greater feelings of T2 self‐help group belonging were related to increased T2 health literacy. Furthermore, health literacy change scores was positively correlated with self‐help group belonging change scores (see Table 4).

**TABLE 4:** Means, standard deviations and bivariate correlations of study variables at and across time 1 and time 2

	Mean	*SD*	T1 SE	T1 SRH	T1 SHGB	T1 HL	T2 SE	T2 SRH	T2 SHGB	T2 HL	HL C
T1 SE	1.52	1.03	—								
T1 SRH	1.31	0.99	0.44**	—							
T1 SHGB	3.82	0.33	−0.02	−0.15	—						
T1 HL	5.72	1.87	−0.43**	−0.49**	0.18	—					
T2 SE	0.69	0.83	0.11	0.17	−0.17	−0.11	—				
T2 SRH	0.58	0.69	0.29*	0.13	−0.15	−0.03	0.51**	—			
T2 SHGB	3.82	0.38	−0.43**	−0.02	0.02	0.13	−0.04	−0.29*	—		
T2 HL	6.48	1.95	−0.17	−0.08	−0.08	0.25*	0.27*	−0.42*	0.27*	—	
HL change	0.64	2.31	0.18	0.29*	−0.31*	−0.59**	−0.31*	−0.34**	0.18	0.64**	—
SHGB change	−0.03	0.48	−0.34**	0.12	−0.62**	0.06	−0.14	−0.31**	0.77**	0.27*	0.35**

Note

*p < 0.05, **p < 0.001.

Abbreviations: *SD*, standard deviation; SE, stereotype endorsement; SRH, stigma‐related harm; SHGB, self‐help group belonging; HL, health literacy; HL Change, health literacy change; SHGB change, self‐help group belonging change.

### 3.4 Health literacy and stereotype endorsement as mediators of relationships between self‐help group belonging and stigma‐related harm

In line with our theoretical position, we used the self‐help group change score as a predictor of health literacy change in the serial mediation model. There was a significant direct effect of self‐help group belonging change on T2 stigma‐related harm, *β* = −0.48, *p* = 0.006. Change in self‐help group belonging also significantly predicted change in health literacy. And change in health literacy significantly predicted T2 stereotype endorsement, which in turn predicted T2 stigma‐related harm. The model of the sequential indirect effect of change in self‐help group belonging on T2 stigma‐related harm via health literacy change and T2 stereotyped endorsement was significant (Indirect effects, *β* = −0.06; 95% CI [−0.18, −0.008]) explaining 35% of the variance in T2 stigma‐related harm and supporting hypothesis 2. And including these two mediators, meant the direct effect was non‐significant (see Table 5).

**TABLE 5:** Serial mediation of change in self‐help group belonging on T2 stigma‐related harm via health literacy change and T2 stereotype endorsement

		Health literacy change				T2 stereotype endorsement				*Y* T2 Stigma‐related harm		
		Coeff	95% CIs	*p*		Coeff	95% CIs	*p*		Coeff	95% CIs	*p*
Antecedent												
Constant		0.65	0.11, 1.19	0.019		0.78	0.57, 0.99	<0.001		0.34	0.14, 0.54	0.001
*X* SHGB change	a_1_	1.68	0.55, 2.81	0.004	a_2_	−0.12	−0.57, 0.33	0.59	c’	−0.30	−0.61, 0.01	0.061
HL Change		—	—	—	d_21_	−0.10	−0.20, −0.01	0.031	b_1_	−0.05	−0.12, 0.02	0.15
T2 Stereotype Endorsement		—	—	—		—	—	—	b_2_	−0.35	0.17, 0.52	<0.001
Model R^2^	R^2^ = 0.12, *F*(1,64) = 8.84, *p* = 0.004				R^2^ = 0.10, *F*(2,63) = 3.43, *p* = 0.039				R^2^ = 0.35, *F*(3,62) = 11.33, *p* < 0.001			
Model controlling for T1 stereotype endorsement and stigma‐related harm												
Constant		−0.49	−1.44, 0.47	0.31		0.62	0.26, 0.99	0.001		0.05	−0.22, 0.31	0.72
*X* SHGB change	a_1_	2.00	0.80, 3.21	0.002	a_2_	0.14	−0.36, 0.63	0.59	c’	−0.25	−0.58, 0.08	0.14
HL change		—	—	—	d_21_	−0.12	−0.22, −0.03	0.013	b_1_	−0.08	−0.15, −0.01	0.020
T2 Stereotype Endorsement		—	—	—		—	—	—	b_2_	0.34	0.17, 0.51	<0.001
T1 Stereotype Endorsement		0.57	0.006, 1.14	0.048		0.24	0.02, 0.46	0.036		0.08	−0.08, 0.23	0.32
T1 Stigma‐related Harm		0.26	−0.35, 0.88	0.40		−0.15	−0.39, 0.09	0.21		0.18	0.02, 0.34	0.030
Model R^2^	R^2^ = 0.22, *F*(3,62) = 5.94, *p* = 0.001				R^2^ = 0.16, *F*(4,61) = 2.98, *p* = 0.026				R^2^ = 0.45, *F*(5,60) = 9.64, *p* < 0.001			

Abbreviations: SHGB change, self‐help group belonging change; HL Change, health literacy change; T1, time 1; T2, time 2.

**FIGURE 1:**
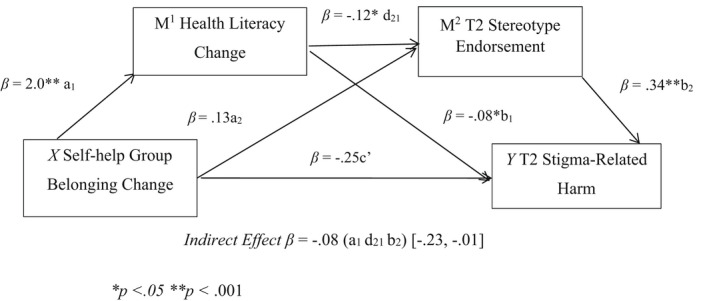
Conceptual Representation of Serial Mediation Model. An outline of the relationship between self‐help group belonging change and lower T2 stigma‐related harm significantly mediated by health literacy change and reduced T2 stereotype endorsement, while controlling for T1 stereotype endorsement and T1 stigma‐related harm

Capitalising on the longitudinal design, we also tested the model whilst controlling for T1 measures of stereotype endorsement and stigma‐related harm. This increased the amount of variance explained by the model to 44%. Further, T1 stereotype endorsement was not a significant predictor of T2 stigma‐related harm, but T1 stigma‐related harm was a significant predictor of T2 stigma‐related harm in the model. This suggests a reasonably robust effect for change in self‐help group belonging, which had a significant sequential indirect effect on T2 stigma‐related harm via health literacy change and T2 stereotype endorsement (Indirect effects, *β* = −0.08; 95% CI [−0.23, −0.01]) when controlling for T1 levels of these variables (see Table 5 and Figure 1).

## 4 DISCUSSION

In a longitudinal study, we assessed the impact of belonging to self‐help groups on reducing stigma associated with leprosy among rural village dwellers in Nepal. Specifically, our hypothesis was that increased self‐help group belonging and increased health literacy reduces stereotype endorsement among those affected by leprosy, which in turn reduces the stigma‐related harm associated with that condition (i.e., an indirect effect). As expected, health literacy significantly increased across time. Furthermore, we found that members of the self‐help groups experienced significantly less stereotype endorsement and stigma‐related harm at time 2. Changes in self‐help group belonging and increased health literacy were associated with reduced stereotype endorsement and stigma‐related harm at time 2 also. As expected, and consistent with the social cure model, we found a significant indirect effect of changed self‐help group belonging on reduced stigma‐related harm via changed health literacy and reduced stereotype endorsement, supporting our main hypothesis. Indirect effects were maintained when we controlled for time 1 levels of these variables.

These findings illustrate the duality of the effects of groups on health. On the one hand, the membership of a self‐help group is related to improved health literacy whilst on the other, membership of a stigmatised illness group and any associated endorsement of leprosy stereotypes is related to more stigma‐related harm. The finding that improved health literacy reduces stigma‐related harm via reduced stereotype endorsement and is consistent with the progressive model of self‐stigma (Corrigan et al., 2011). This model presupposes that people affected by a stigmatised condition are aware of the stereotype relating to their group, may then accept it, apply it to the self and suffer harm such as feelings of shame and reduced self‐esteem. A key tenet of the progressive model of self‐stigma (Corrigan et al., 2011) is that the stereotype endorsement stage is a proximal aspect of self‐stigma. A key contribution of our research is that it highlights how a social cure intervention to reduce stereotype endorsement by increasing health literacy can mitigate some of the harm associated with the self‐stigma of leprosy. Importantly our findings suggest that membership of these self‐help groups not only have important positive consequences for health literacy but also for the feelings of stigma and associated mental health consequence of this stigma. As such we believe this approach is an important antidote to the social exclusion and mental health costs of leprosy. Though this is beyond the scope of the current project, it is likely that this model and our findings have implications for hard to reach and very under‐represented populations similarly affected by other infectious‐stigmatised diseases. This is an important avenue for future research.

Currently there is a critical need for theoretically grounded, community health and social care interventions to address the myriad of issues associated with health generally, and leprosy in particular, in real world and non‐WEIRD settings. Nepal is a case in point. Our findings highlight that specific essential services tackling leprosy are effective in driving down stigma in the community as well as offering improved health literacy through community services. NLT as an authorised Government service is now ideally placed to support and inform the National Leprosy Control Programme. This community‐based approach is an important element that can strengthen the leprosy services. NLT is already tasked with training and capacity Building of government health staff. This community‐based approach to management of leprosy is not only cost efficient, but also offers a valuable approach to reducing the human costs of leprosy through improved self‐management and treatment of leprosy. It also has the power to destigmatise leprosy, which will ultimately assist in early detection and prevention.

We believe that the integration of the two models contributes to understanding of the seemingly contradictory influences of group memberships and social identities on health. Our research approach is grounded in an applied perspective from within social psychology (Haslam, 2014)), which parallels with realist evaluation approach (Dalkin et al., 2012). Although randomised controlled designs have well‐established empirical rigor and are considered the gold standard for evaluation of intervention efficacy (O'Shea et al., 2016), previous researchers have expressed some doubts regarding the utility of this particular paradigm as an effective framework for evaluating interventions in applied domains. One popular objection to these trials focuses on the allocation of a control group as inherently unethical and exploitative in nature. This position is particularly relevant given the highly unique contextual and procedural issues within the current research, where the self‐help group intervention was highly likely to be of extreme value to members. As with all community‐engaged research, this required critical examination between collaborators around the range of risks and issues that may arise. As such, deliberately withholding this intervention for the sake of stronger empirical validity was considered unethical, and the duty of care to the participants took precedence in the current design. Indeed, as discussed earlier, substantial time and resources were devoted to training workshops addressing various power imbalances, and safeguards to ensure the moral and ethical sensitivity of this research. To offer no support to those affected by Leprosy, when supports are available, would erode developing trust in both health practitioners and researchers in marginalised and very vulnerable communities. The current study employed a longitudinal design, which allowed us to demonstrate that self‐help group members experienced improvements in health literacy and reductions in stereotype endorsement and stigma‐related harm over the 6 month period. There were no significant differences in self‐help group belonging across the 6 month period. This is likely due to the large concentration of scores at the upper and limits of these measures. This may be accounted for by considering the socio‐political context and cultural milieu of the participants. These individuals are among the most marginalised, disadvantaged and poor members of Nepali society, and are affected by one of the most stigmatised diseases world wide (Stevelink et al., 2011; Weiss, 2008). As such, they may be extremely grateful to be involved in the self‐help groups, where they learn to support and care for each other and often become collectively empowered as a result (Cross et al., 2017). It may also point to the complexities around translation and cross‐cultural adaptation and validation of instruments, and the attrition in our sample not least because we were dealing with non‐WEIRD people who migrate to work and survive.

Nonetheless support for our two models is extremely encouraging. These findings provide preliminary evidence in favour of utilising the self‐help group approach to target both psychosocial and physical health, to enhance the education and health of the very poor, even where some of the population may be transient and communities in flux. Of particular importance is the evidence that the current self‐help group, through improved health literacy, appears to ameliorate some of the negative internalisations of leprosy stigma. Given that leprosy is arguably one of the most chronically stigmatised diseases world wide (Stevelink et al., 2011), the present findings highlight the power and potential of a self‐help group approach to facilitate change in the lived experiences of those affected. This includes mental and physical health benefits. In addition, these findings contribute to, and are consistent with, the accumulating evidence in support of how social curative identity resources can be used to overcome the harmful effects of stigmatised identities across different social and cultural contexts. Moreover, this was accomplished using a non‐WEIRD sample, avoiding the current bias and cross cultural generalisability assumptions that are argued to have compromised the social sciences as a whole (Henrich et al., 2010). Given the lack of theoretically driven and community engaged interventions to combat the innumerable issues associated with stigma in the world's most deprived populations, this line of inquiry can be seen as both timely and promising. Investigating the tenets of the progressive model of self‐stigma and the social identity approach together in this applied, community context, is a distinct step in the desired direction. The empirical support for the integration of these ideas represents a novel contribution to the literature.

## AUTHOR CONTRIBUTION

OM, MW secured research funding; OM, MW, SJ, AO'D, BO'C, RC, KJ and AKS developed research protocol and materials; RC, KJ and AKS managed data collection; OM, SJ, MW, RC, KJ and AKS trained research team and completed data collection, AM. BO'C completed data analysis and drafted results; OM, SJ and AO'D drafted the paper; All authors inputted and reviewed drafts and revisions.

## Data Availability

The data that support the findings of this study are available upon request from the corresponding author [OM]. The data are not publicly available because of the information contained, which could compromise the privacy of research participants.
